# Type 1 neovascularization may confer resistance to geographic atrophy amongst eyes treated for neovascular age-related macular degeneration

**DOI:** 10.1186/s40942-015-0015-6

**Published:** 2015-09-03

**Authors:** Elona Dhrami-Gavazi, Chandrakumar Balaratnasingam, Winston Lee, K. Bailey Freund

**Affiliations:** 1 Vitreous Retina Macula Consultants of New York, 460 Park Avenue, 5th Floor, New York, NY 10022 USA; 2 The LuEsther T. Mertz Retinal Research Center, Manhattan Eye Ear and Throat Hospital, New York, NY USA; 3 Department of Ophthalmology, Edward S. Harkness Eye Institute, Columbia University, College of Physicians and Surgeons, New York, NY USA; 4 Department of Ophthalmology, New York University School of Medicine, New York, NY USA

**Keywords:** Age-related macular degeneration, Geographic atrophy, Type 1 neovascularization, Anti-vascular endothelial growth factor

## Abstract

**Background:**

To report a series of age-related macular degeneration (AMD) patients in whom progression to geographic atrophy (GA) in one eye receiving frequent intravitreal injections of anti-vascular endothelial growth factor (anti-VEGF) therapy for type 1 neovascularization (NV) was slower than that of the fellow eye with non-neovascular AMD.

**Methods:**

Retrospective, observational case series examining the clinical course and GA progression rate in four consecutive patients in which one eye harbored type 1 neovascular AMD and was receiving anti-VEGF therapy, while the fellow eye manifested signs of non-neovascular AMD only. Eligibility criteria included anti-VEGF therapy duration of over 4 years and over 50 injections. Lesion evolution was documented via multimodal imaging. GA at baseline and final visits was quantified and GA progression rate for each eye was determined.

**Results:**

Four consecutive patients were followed for a mean interval of 94 months (range 62–120). One eye harbored type 1 NV while the fellow eye remained non-neovascular. The former received a mean of 65.5 ± 15.2 anti-VEGF injections. Mean rate of GA progression in non-neovascular eyes was 0.076 ± 0.024 mm^2^/month and in type 1 NV eyes was 0.004 ± 0.005 mm^2^/month. Difference in GA progression rate between type 1 and non-neovascular eyes was found to be statistically significant (P = 0.001).

**Conclusions:**

These findings support previous hypotheses that, unlike type 2 and 3 lesions, type 1 NV may represent a neovascular AMD subtype more resilient to GA formation. This may have implications for anti-VEGF regimens in the management of type 1 NV.

## Background

Geographic atrophy (GA) is a form of advanced age-related macular degeneration (AMD) and is a term that is used to denote the occurrence of retinal pigment epithelium (RPE), photoreceptor and choriocapillaris loss [1, 2]. Central GA, with resultant disruption of foveal architecture, is typically characterized by poor visual function [3]. There is great interest in the pathophysiological mechanisms that govern the rate of GA progression as well as the iatrogenic and treatment-induced factors that accelerate or retard its progression. With regard to non-neovascular AMD, drusen characteristics have been used to stratify the risk of GA development [4]. In the neovascular form of AMD, the subtype of neovascularization [5, 6] classified with either fluorescein angiography (FA) alone [as poorly defined (occult), well-defined (classic) or as retinal angiomatous proliferation (RAP)] or anatomically using both FA and OCT (as type 1—sub retinal pigment epithelium, type 2—subretinal, or type 3—intraretinal) is believed to influence the risk of GA progression [7].

Recently, results from the comparison of AMD treatment trials (CATT) study and Inhibition of VEGF in age-related choroidal neovascularization (IVAN) trial have raised some concerns regarding the effects of long-term anti-vascular endothelial growth factor (VEGF) therapy on the outer retina [8]. The results from both of these studies have suggested that the duration and frequency of anti-VEGF therapy may be related to the risk of new GA and GA progression. One way by which long-term anti-VEGF therapy may accelerate the rate of GA progression is by nullifying VEGF activity in the outer retina beyond the physiological concentrations that are required for homeostatic function. Causal relationships between GA and anti-VEGF therapy demonstrated in the CATT and IVAN studies have been supported by experimental work in mice where the absence of soluble VEGF isoforms resulted in the development of choroidal atrophy and attenuation/loss of RPE [9]. The net effect of these experimental changes was photoreceptor death and a decline in visual function [9]. The risk of GA atrophy following anti-VEGF therapy is, however, not independent of NV subtype. In a recent study by Xu et al. [7], it was shown that eyes harboring type 1 neovascularization (NV) were associated with a relatively lower risk of GA formation and progression. In contrast, type 3 NV eyes were at a considerably higher risk of GA formation. Another study by Chae et al., also found that eyes with neovascular AMD that had type 1 NV at baseline, had a more favorable long-term visual prognosis on a treat-and-extend regimen of anti-VEGF agents [10]. Expanding our understanding about the clinical and multimodal imaging (MMI) characteristics of NV subtypes and the relationships that they bear to the development of GA is therefore likely to aid with a more individualized patient management.

In this report we describe the clinical and MMI characteristics of 4 patients who developed a greater degree of GA in the eye that manifested signs of non-neovascular AMD relative to the contralateral eye that was receiving frequent anti-VEGF injections for type 1 NV. These findings are used to speculate upon the potentially protective role of sub-RPE neovascular tissue in reducing the rate of GA progression.

## Methods

This retrospective cohort study design was approved by the Western Institutional Review Board (Olympia, WA, USA). It complied with the Health Insurance Portability and Accountability Act of 1996 and followed the Tenets of the Declaration of Helsinki.

Consecutive patients with neovascular AMD that met the eligibility criteria were enrolled into the study in a retrospective manner. Patients included in this study met all of the following inclusion criteria: (1) Frequent and continuous anti-VEGF treatment for type 1 NV was being administered to one eye for 4 or more years. (2) 50 or more injections of anti-VEGF therapy had been administered to the eye with type 1 NV. (3) The eye receiving anti-VEGF therapy was treatment-naïve at the initiation of treatment and had not received steroids, laser or other treatments. (4) The fellow eye manifested signs of non-neovascular AMD for the duration of the study. Exclusionary criterion was the presence of neovascular AMD in the fellow eye.

All patients were followed and treated by a single retina specialist (KBF) between January 2004 and January 2015. However, the date of the first injection was considered the baseline study visit. The following data were recorded from each patient: age, gender, ocular and systemic comorbidities, concurrent medications, type and number of anti-VEGF injections administered to the eye with type 1 NV, interval at which they were treated, and best corrected Snellen visual acuity at baseline and at last follow-up to-date (using the patient’s current spectacles with and without pinholes). Multimodal imaging results including color photographs, fluorescein angiography (FA), fundus autofluorescence (AF), near-infrared reflectance (IR) and spectral-domain optical coherence tomography (SD-OCT) were reviewed. The anatomical classification of the neovascular lesion was determined using FA and SD-OCT by an unmasked, independent grader. Wide field (488 nm) AF and color fundus images were acquired with the TRC-501X fundus camera (Topcon Imagenet, Tokyo, Japan). Thus, all GA measurements were done through the same software. SD-OCT and IR images were obtained using the Heidelberg Spectralis HRA + OCT (Heidelberg Engineering, Inc, Vista, CA, USA). The AutoRescan function of the Spectralis platform permitted accurate alignment of SD-OCT scans between visits and facilitated reliable longitudinal comparisons. The Heidelberg Spectralis HRA + OCT permitted AF or IR images to be simultaneously obtained with registered eye-tracking and thus facilitated reliable comparisons between baseline and follow-up visits. The areas of GA formation at baseline and last follow-up were outlined in each of the eight eyes of the four patients. Progression of GA was defined as de novo development of atrophy or an enlargement of the preexisting area(s) of GA (dark pixels) compared to baseline. Measurements of the total area of regions of GA were made and assessed manually on scaled AF images from the Topcon IMAGEnet i-base software. The differences in total GA area between the last visit and the baseline were calculated and the rate of progression was determined by dividing the difference in GA size (in mm^2^ units) by the time between the baseline and last visits (expressed in months). In eyes with type 1 NV, the rate of progression was standardized to the number of injections received by dividing the difference in GA size between baseline and final visits by the total number of injections administered. A two-tailed *t* test was used to determine if the rate of GA progression (expressed in mm^2^/month) was different between the eye with non-neovascular manifestations and the eye with type 1 NV receiving anti-VEGF therapy. The outlining of GA lesion edges for qualitative analyses and figure preparation was conducted in Photoshop CS3 (Adobe, San Jose, CA, USA) by pixel threshold intensity segmentation.

## Results

### General

The mean follow-up interval for 4 patients (1 male and 3 female) with a mean age of 85.5 years (range 76–95) was 93.75 months (range 64–120). The mean number of anti-VEGF injections administered to the eye with type 1 NV was 65.5 ± 15.2. The mean rate of GA progression in the non-neovascular eye was 0.076 ± 0.024 mm^2^/month and in the eye with type 1 NV was 0.004 ± 0.005 mm^2^/month. The difference in the rate of GA progression between neovascular and non-neovascular eyes was found to be statistically significant (*P* = 0.001).

**Fig. 1:**
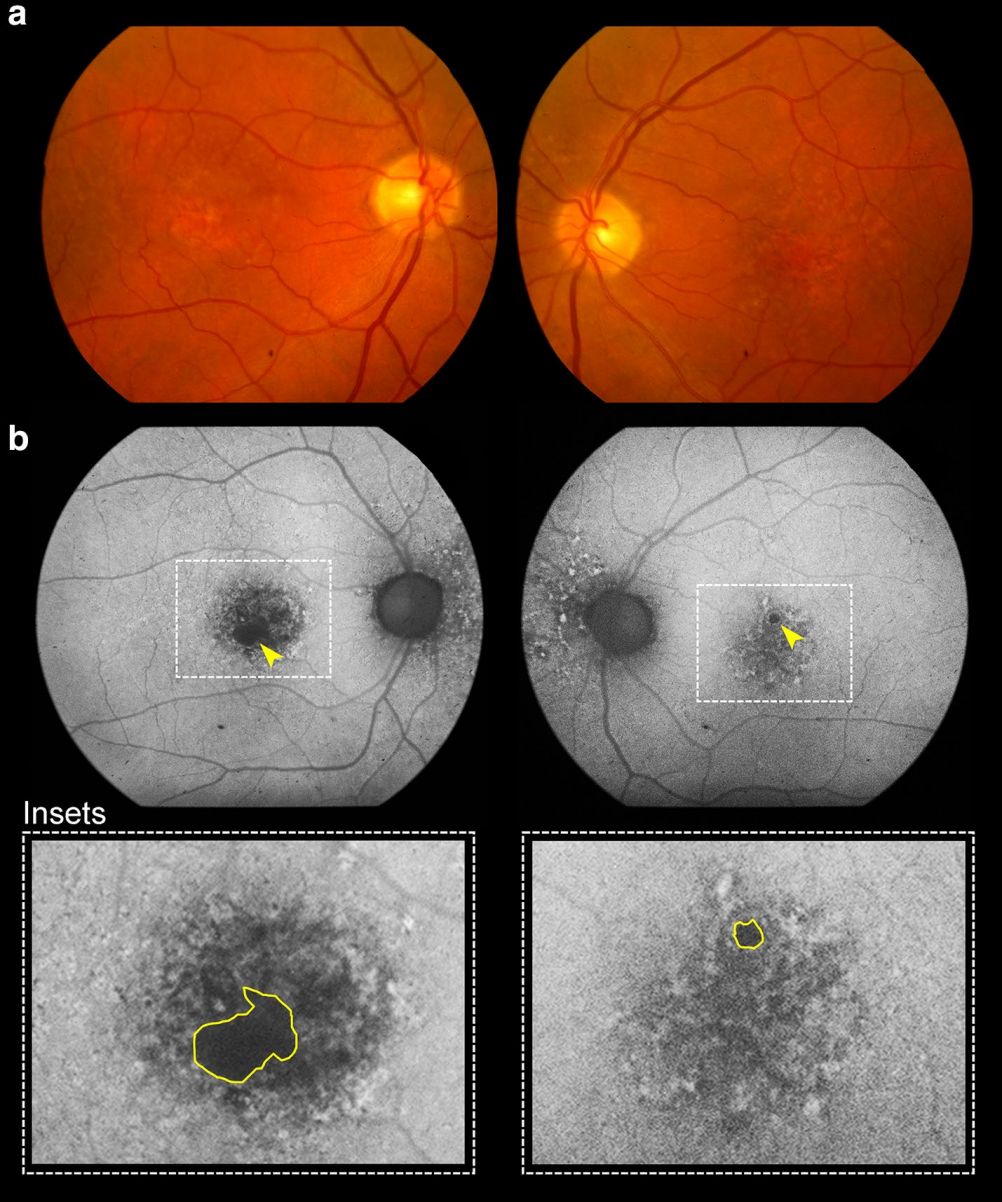
Patient 1 at presentation. Color fundus (**a**) and fundus autofluorescence images (**b**) demonstrate geographic atrophy at the infero-temporal aspect of the right macula and a small atrophic spot at the superior aspect of the left macula (*arrowheads*). Speckled areas of hyper- and hypo-autofluorescence can also be seen at the nasal aspect of the optic discs bilaterally. *Insets* provide high magnification views of the macula where regions of atrophy have been demarcated (*yellow lines*)

**Fig. 2:**
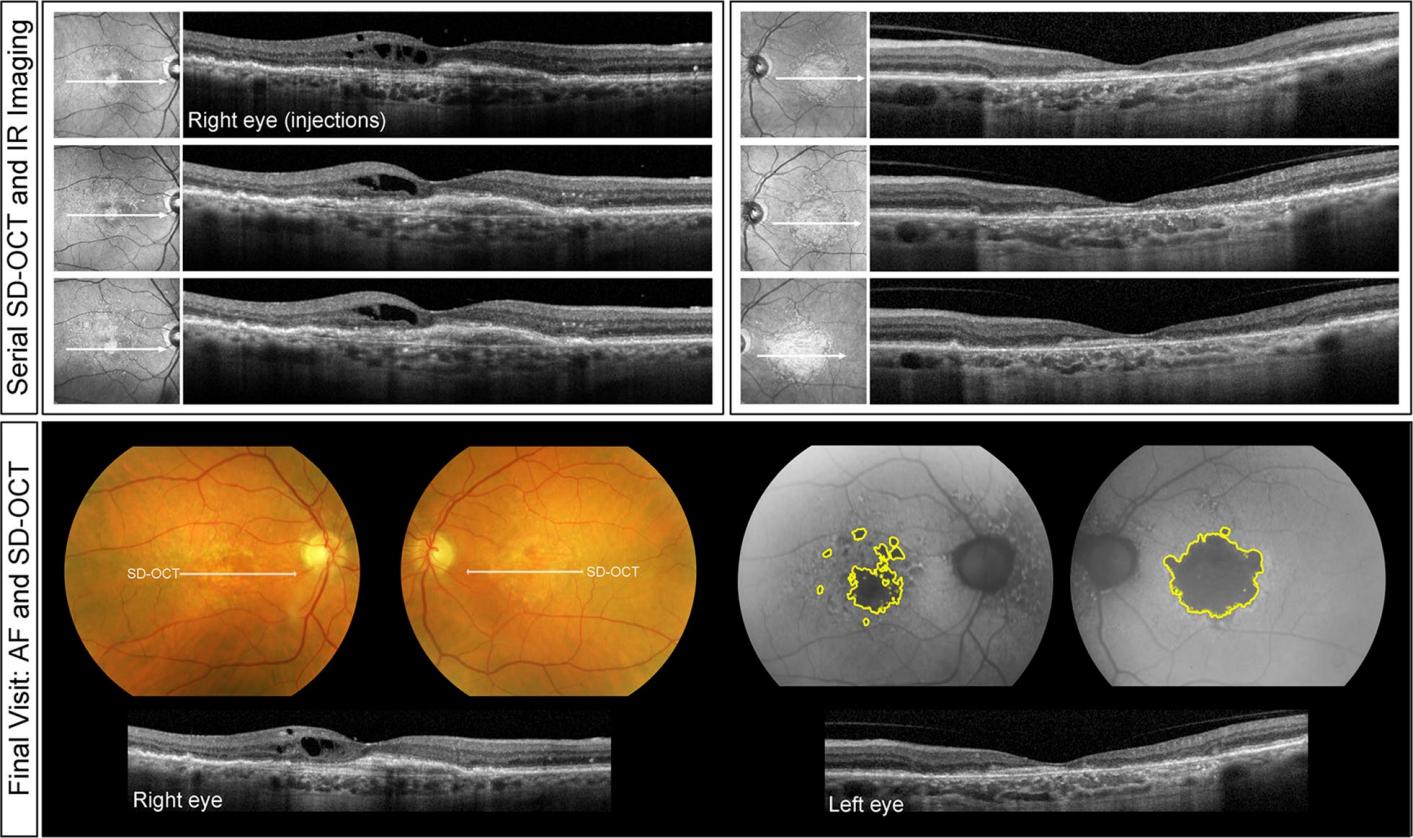
Clinical course of Patient 1. Serial spectral-domain optical coherence tomography (SD-OCT) images taken approximately every 2 years demonstrate relative preservation of outer retinal structures in the right eye (*left panel*) and progressive atrophy in the left eye (*right panel*). Color fundus and fundus autofluorescence images acquired at the final visit demonstrate a greater area of atrophy in the left eye relative to the right. SD-OCT images of the final visit reveal persistent intraretinal edema from type 1 NV in the right eye

**Fig. 3:**
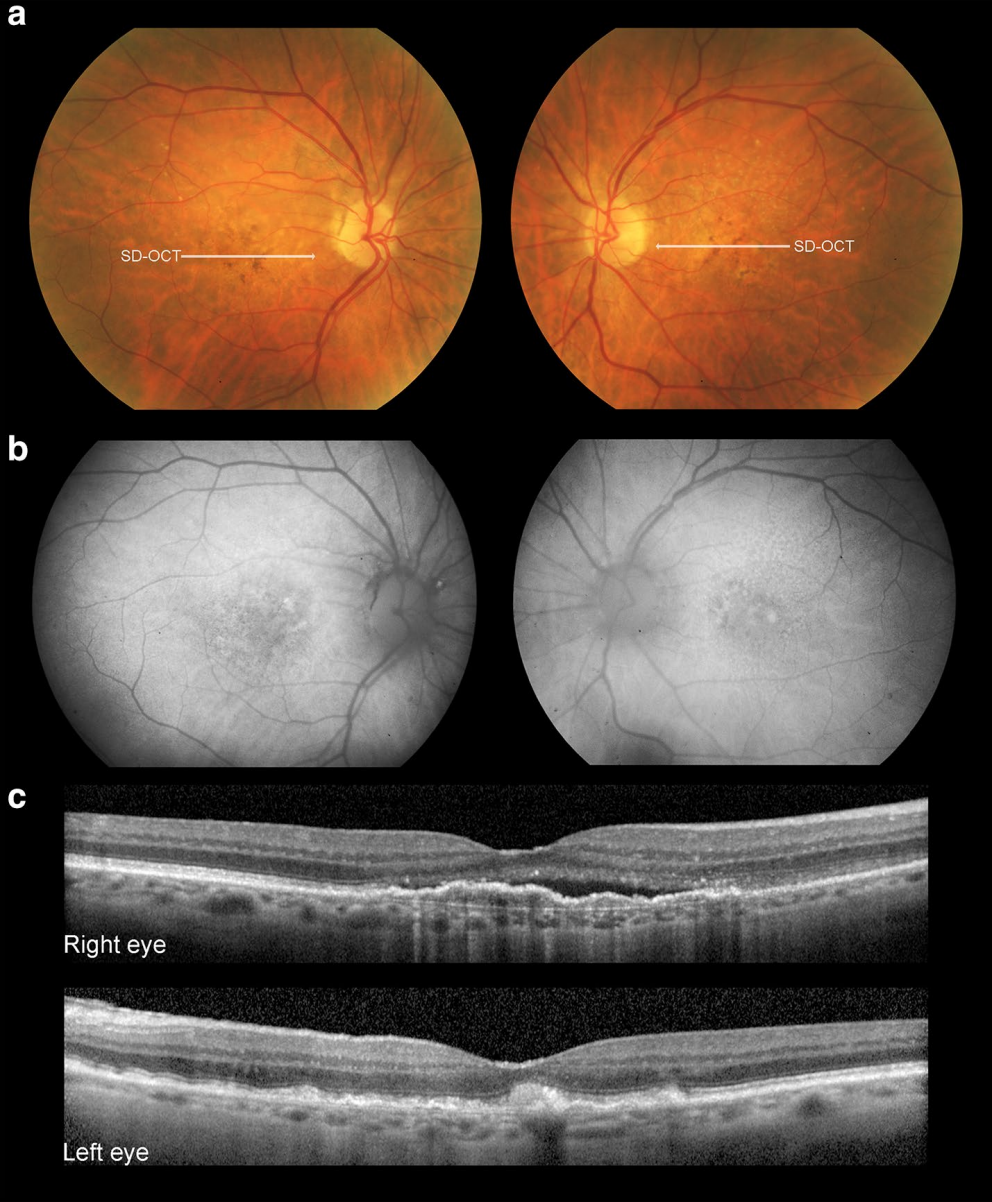
Patient 2 at presentation. Color fundus (**a**) and fundus autofluorescence (**b**) images demonstrate the absence of geographic atrophy at the baseline visit. Reticular pseudodrusen and mottled retinal pigment epithelial changes are evident bilaterally. Spectral-domain optical coherence tomography images (**c**) demonstrate subretinal fluid and type 1 neovascularization in the right eye. A vitelliform lesion is present in the left fovea

**Fig. 4:**
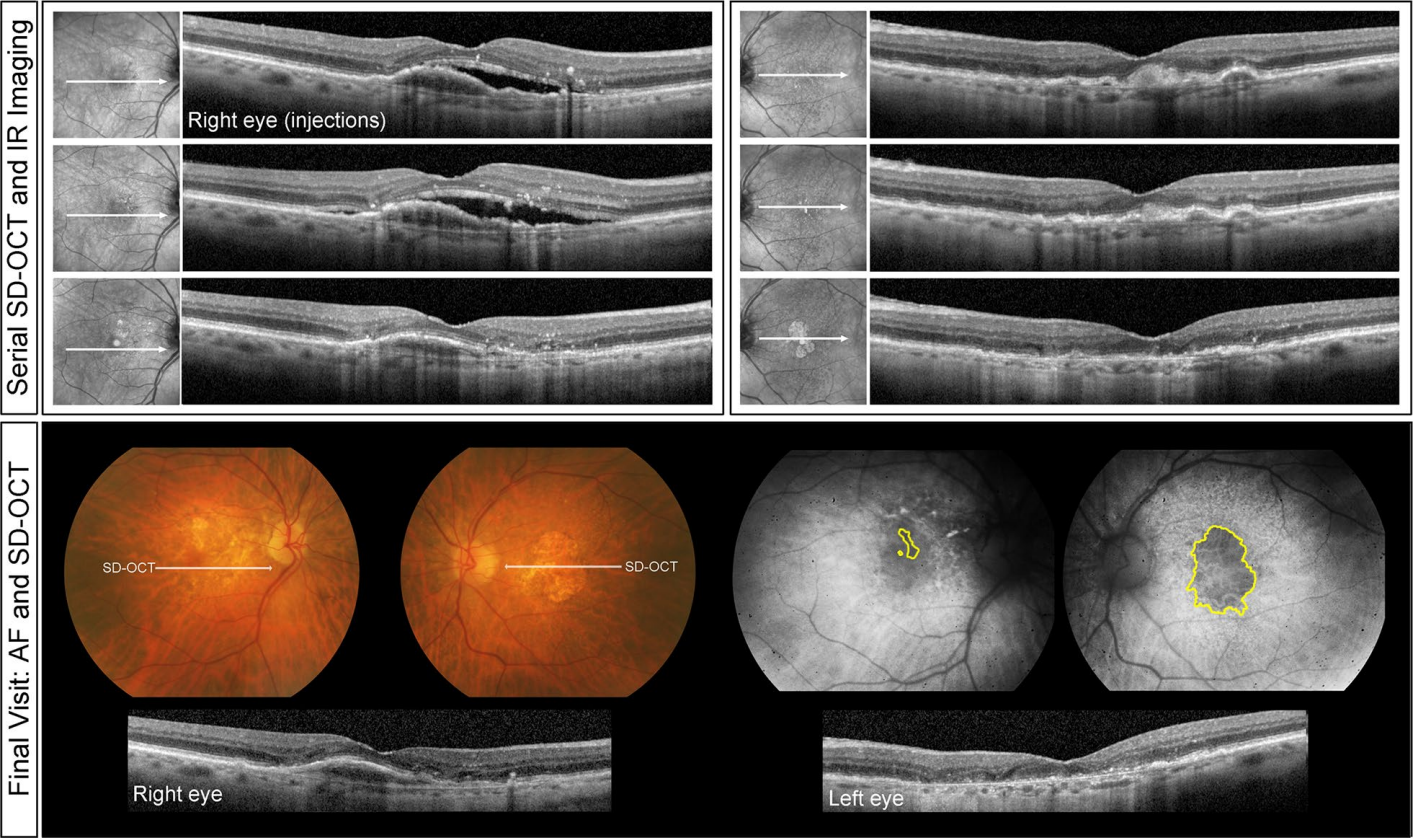
Clinical course of Patient 2. Serial spectral-domain optical coherence tomography (SD-OCT) images taken approximately every 2 years demonstrate resolution of subretinal fluid and preservation of outer retinal structures in the right eye (*left panel*) and reabsorption of vitelliform material, thinning of the choriocapillaris, retinal pigment epitheium and photoreceptor layer in the left eye (*right panel*). Color fundus and fundus autofluorescence images acquired at the final visit demonstrate a greater area of atrophy in the left eye relative to the right. SD-OCT images of the final visit reveal persistent type 1 NV in the right eye

**Fig. 5:**
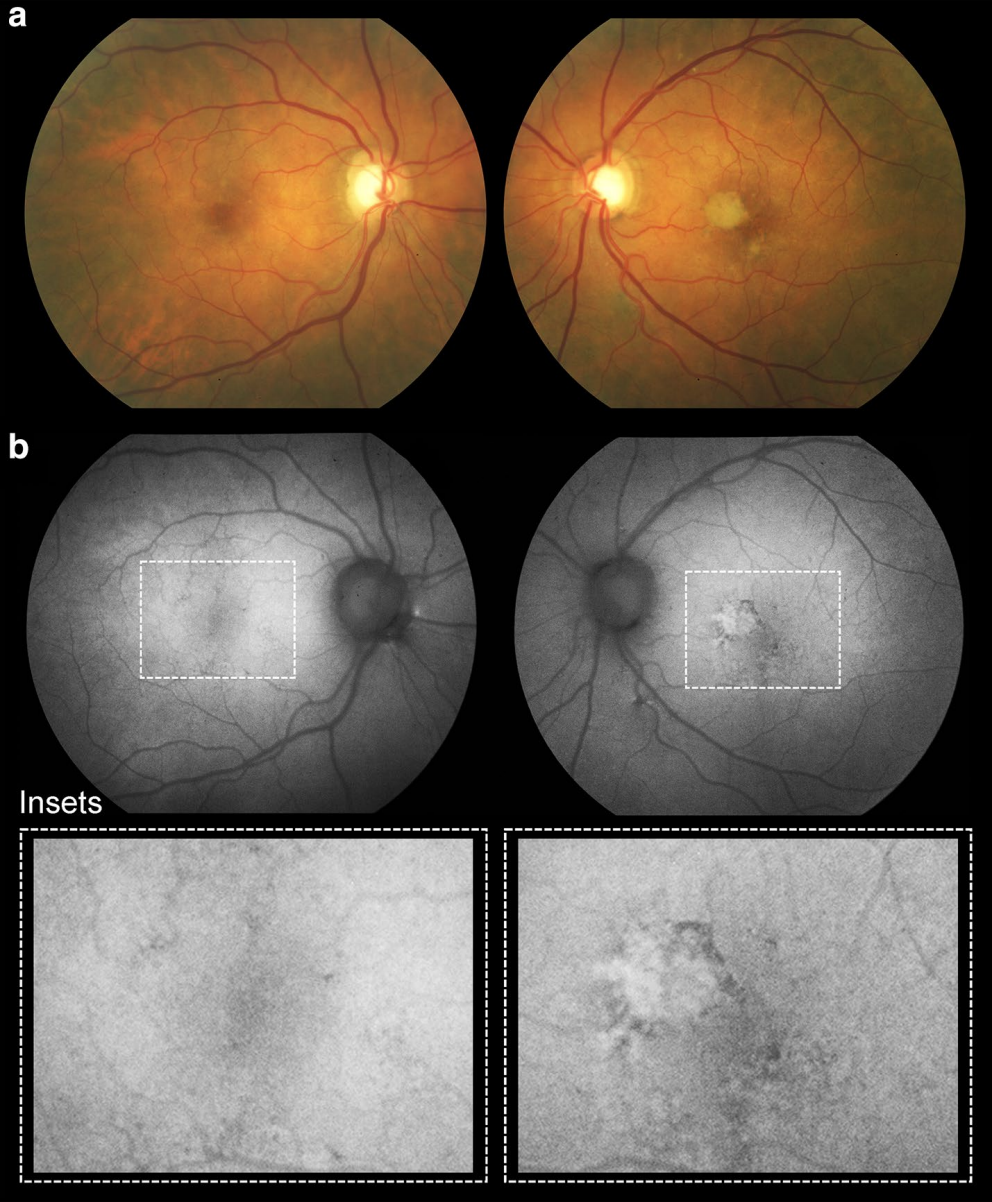
Patient 3 at presentation. As noted on color photographs (**a**) and fundus autofluorescence (**b**), neither eye had geographic atrophy. Mild non-neovascular AMD changes consisting of drusen and pigment mottling were present in the right eye while a juxtafoveal acquired vitelliform lesion was present in the left eye, in addition to somewhat denser drusen and pigment clumping

**Fig. 6:**
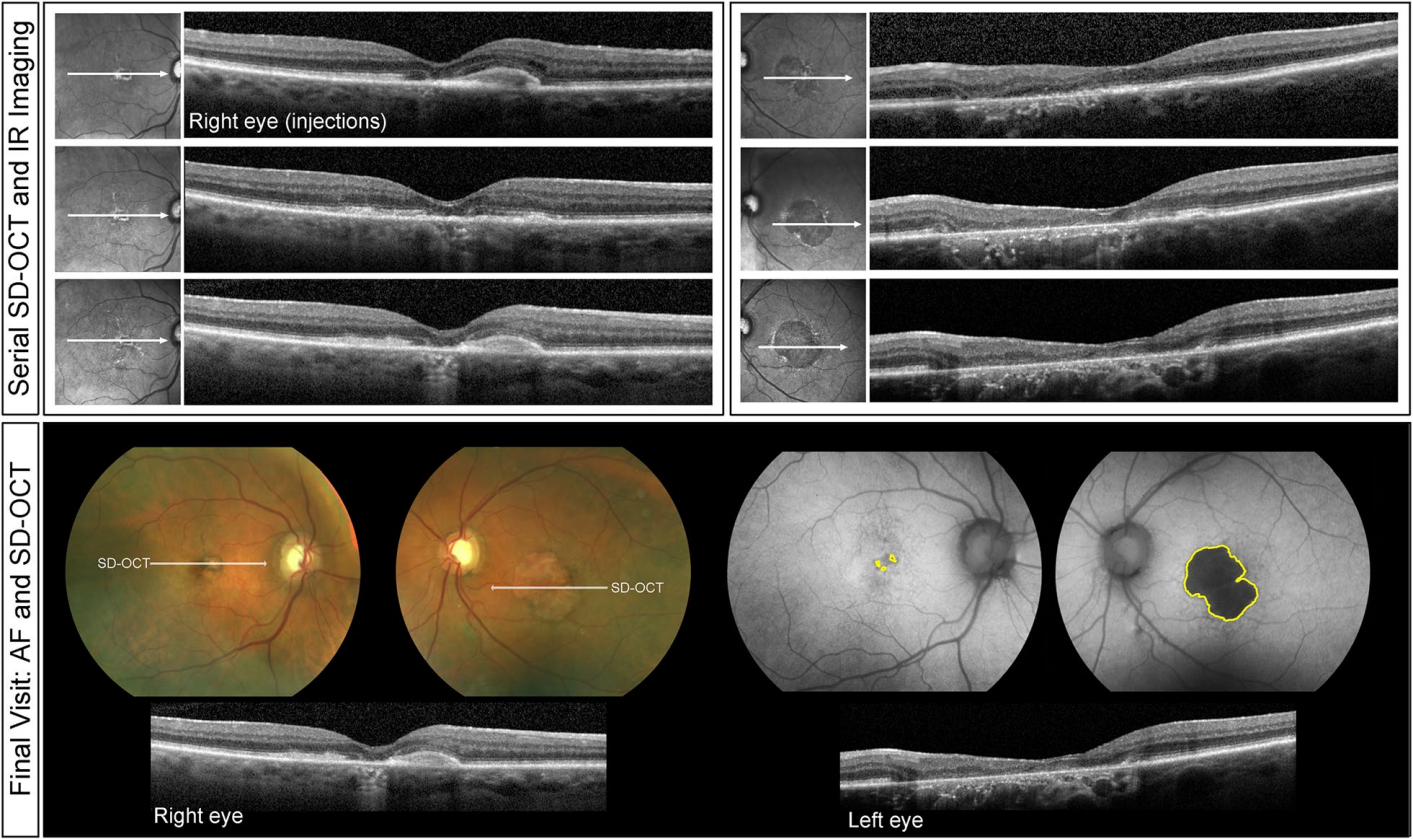
Clinical course of Patient 3. Serial spectral-domain optical coherence tomography (SD-OCT) images taken approximately every 2 years demonstrate gradual progression of central geographic atrophy in the left eye (*right panel*) and minimal progression of foveal atrophy in the right eye (*left panel*). The marked difference between the left and right eyes with respect to the area of atrophy (*yellow lines*) is illustrated in color fundus and fundus autofluorescence images that were acquired at the most recent visit. SD-OCT of the right eye at the final visit reveals persistent type 1 NV

**Fig. 7:**
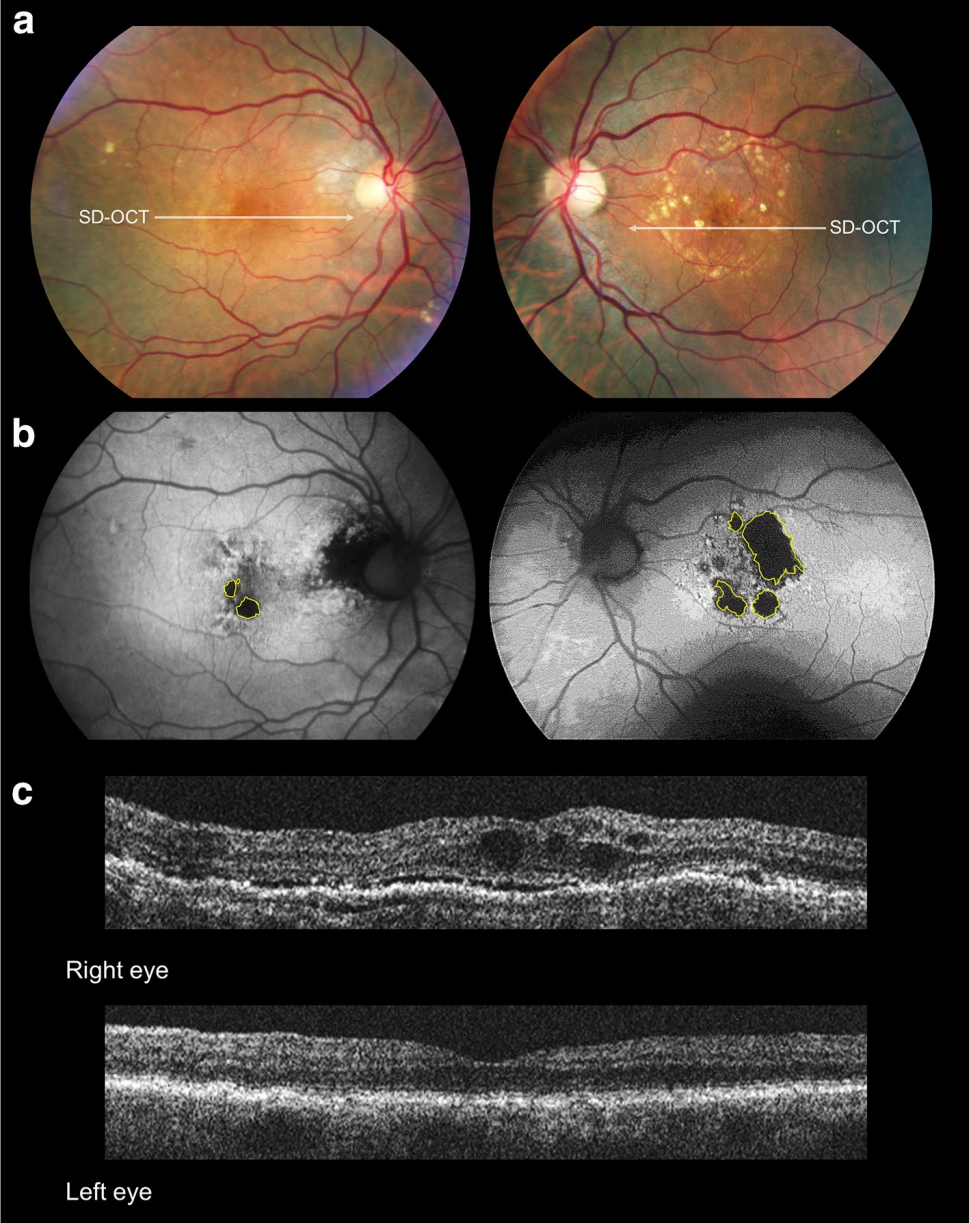
Patient 4 at presentation. Areas of geographic atrophy (*yellow lines*) are clearly evident on color fundus (**a**) and fundus autofluorescence images (**b**) of the left and right eyes. Spectral-domain optical coherence (SD-OCT) images (**c**) taken of the right eye (top) and left eye (bottom) at the initiation of anti-vascular endothelial therapy for the right eye. The scans show subretinal and intraretinal fluid in the right eye and normal subfoveal findings in the left eye

**Fig. 8:**
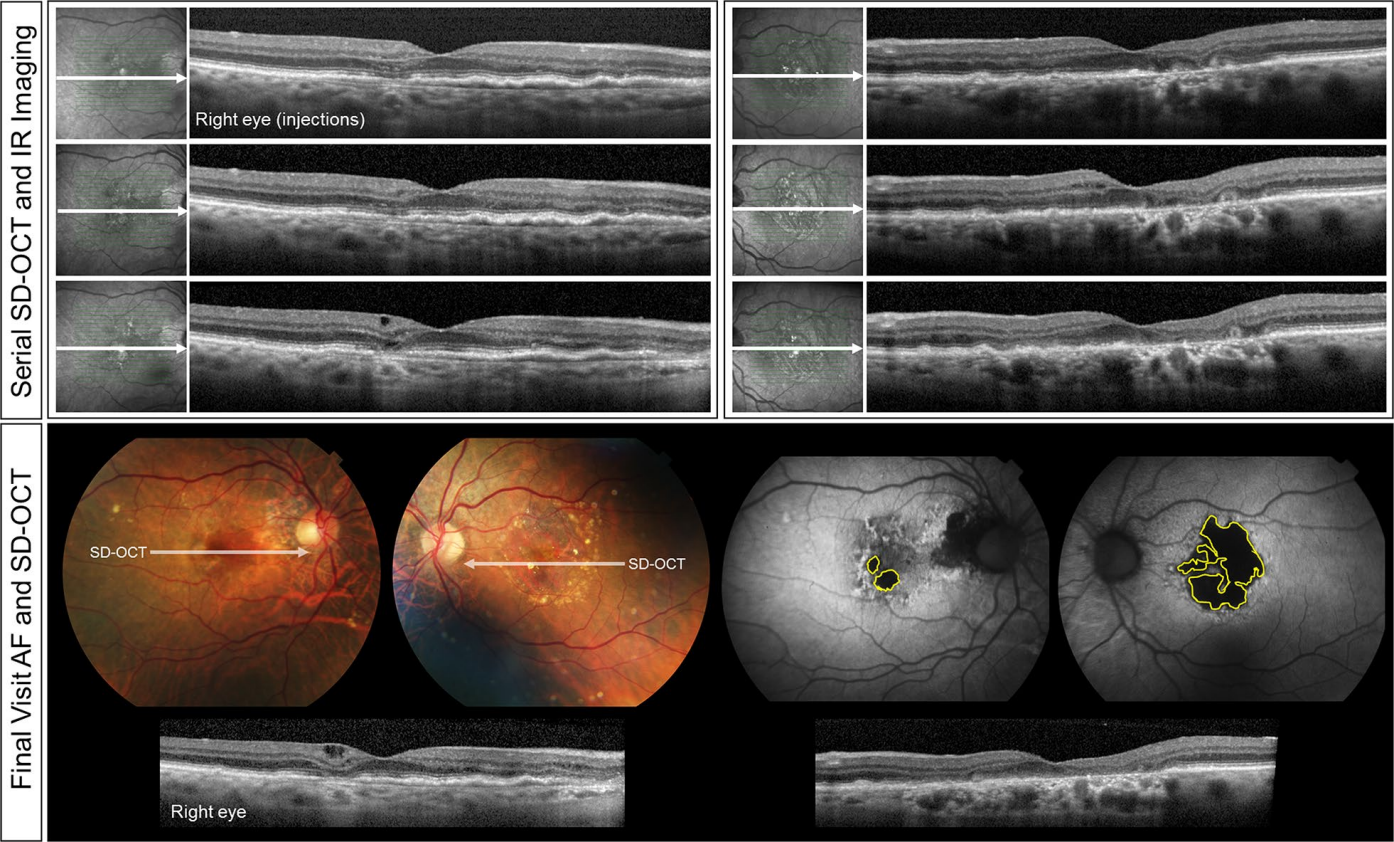
Clinical course of Patient 4. Serial spectral-domain optical coherence tomography (SD-OCT) images taken approximately every 2 years demonstrate gradual progression of central geographic atrophy and the development of outer retinal tubulations in the left eye (*right panel*). In the right eye (*left panel*), there is broad type 1 NV. Color fundus and fundus autofluorescence images acquired at the final visit demonstrate a greater area of atrophy (*yellow lines*) in the left eye relative to the right. SD-OCT image of the final visit also reveals persistent type 1 NV in the right eye with intraretinal edema

The demographics, features of treatment regimen and progression characteristics for GA in each eye are provided in Table 1. In all four patients, the right eye was receiving treatment with anti-VEGF for type 1 NV and the left eye expressed manifestations of non-neovascular AMD. MMI findings for each of the patients in this study are provided in Figs. 1, 2, 3, 4, 5, 6, 7 and 8. Detailed case descriptions for each patient are provided below.

**Table 1:** Summary of patient demographic data, lesion type, treatment regimen and geographic atrophy progression

Eye	Patient 1 (77, WM)		Patient 2 (76, WF)		Patient 3 (94, WF)		Patient 4 (95, WF)	
	OD	OS	OD	OS	OD	OS	OD	OS
Injections received	81 ranibizumab	–	18 ranibizumab; 36 aflibercept	–	51 ranibizumab	–	6 bevacizumab; 70 ranibizumab	–
NV	Type 1	–	Type 1	–	Type 1	–	Type 1	–
Regimen schedule	*q*6 weeks	–	*q*6–7 weeks	–	*q*7–8 weeks	–	*q*4–5 weeks	–
Total follow-up interval	120 months		62 months		99 months		95 months	
Initial visit (mm^2^)	0.38	0.05	0.00	0.00	0.00	0.00	0.56	4.62
Last visit (mm^2^)	1.73	9.65	0.30	6.72	0.07	5.72	0.61	7.84
ΔGA _Last − Initial_	1.35	9.60	0.30	6.72	0.07	5.72	0.05	3.22
Progression rate 1 of GA (mm^2^/month)	0.01125	0.08	0.00483	0.10838	0.00070	0.05777	0.00052	0.03389
Progression rate 2 of GA (mm^2^/injection)	0.01666	–	0.00555	–	0.00137	–	0.00065	–

*WM* white male, *WF* white female, *OD* right eye, *OS* left eye, *CNV* choroidal neovascularization, *mm* ^*2*^ millimeter^2^, *ΔGA* change in geographic atrophy

### Case 1

A 77-year-old white male with a history of hypertension, hyperlipidemia and benign prostate hyperplasia was started on intravitreal anti-VEGF therapy for active type 1 NV in his right eye. His ocular comorbidities included pseudoexfoliation syndrome, mild nuclear sclerosis cataracts, posterior vitreous detachment bilaterally and a choroidal nevus in the nasal retina. Visual acuities at baseline were 20/30 + 2 in the right eye and 20/40 in the left eye. Results of MMI on the initial visit are provided in Fig. 1. Of note, the right eye presented with an area of juxtafoveal GA which was at least five times larger than the spot of GA observed superior to the fovea in the left eye. Type 1 NV developed in the right eye and was treated with 81 intravitreal anti-VEGF injections over the subsequent 120 months on a treat-and-extend-regimen (TER). Injections were given approximately every 5–7 weeks. At the most recent follow-up, visual acuity was 20/50 + 2 in the right eye and 20/400 in the left eye (Fig. 2). The area of GA at baseline visit was 0.38 mm^2^ for the right eye and 0.05 mm^2^ for the left eye. The area of GA at final visit was 1.73 mm^2^ for the right eye and 9.65 mm^2^ for the left eye. The rate of GA progression was 0.01125 mm^2^/month (or 0.01666 mm^2^/anti-VEGF injection) for the right eye and 0.08 mm^2^/month for the left eye.

### Case 2

A 76-year-old white female with a history of hypertension and hyperlipidemia was started on intravitreal anti-VEGF therapy for active type 1 NV in her right eye. Her ocular comorbidities included bilateral retinal breaks treated with laser retinopexy, bilateral narrow anterior chamber angles treated with laser iridotomies, and bilateral mild nuclear sclerotic cataracts. Visual acuities at baseline were 20/25 + 2 in the right eye and 20/25 in the left eye. The baseline MMI of the right eye, including FA (not shown) demonstrated type 1 NV with overlying subretinal fluid. In the left eye there were drusen, reticular pseudodrusen, and a small subfoveal acquired vitelliform lesion (Fig. 3). Neither eye manifested GA at baseline. Over the following 62 months, the right eye received 54 intravitreal anti-VEGF injections on a TER with injections given approximately every 5–7 weeks. At the most recent follow-up, visual acuity was 20/30 − 2 in the right eye and 20/200 in the left eye. The area of GA at baseline visit was 0 mm^2^ for both eyes. The area of GA at final visit was 0.30 mm^2^ for the right eye and 6.72 mm^2^ for the left eye (Fig. 4). The rate of GA progression was 0.00483 mm^2^/month (or 0.00555 mm^2^/anti-VEGF injection) for the right eye and 0.10838 mm^2^/month for the left eye.

### Case 3

A 94-year-old white female with a history of hypertension and spinal stenosis was started on intravitreal anti-VEGF therapy for active type 1 NV in her right eye. Her ocular comorbidities included primary open angle glaucoma that was managed with latanoprost drops in both eyes. The patient was pseudophakic and had mild epiretinal membranes bilaterally. Visual acuities at baseline were 20/30 in each eye. At baseline the right eye demonstrated type 1 neovascular AMD and the left eye had an acquired, juxtafoveal vitelliform lesion in addition to drusen and pigment clumping (Fig. 5). FA (not shown) demonstrated type 1 NV with overlying subretinal fluid. Neither eye manifested GA at baseline. Over the following 99 months, the right eye received 51 intravitreal anti-VEGF injections on a TER with injections given approximately every 6–7 weeks. At the final visit of this study, the BCVA was 20/60 − 2 in the right eye and 20/200 in the left eye. The area of GA at baseline visit was 0 mm^2^ for both eyes. The area of GA at final visit was 0.07 mm^2^ for the right eye and 5.72 mm^2^ for the left eye (Fig. 6). The rate of GA progression was 0.00070 mm^2^/month (or 0.00137 mm^2^/anti-VEGF injection) for the right eye and 0.05777 mm^2^/month for the left eye.

### Case 4

A 95-year-old white female with a history of hypertension was commenced on intravitreal anti-VEGF therapy for active type 1 NV in her right eye. Her ocular comorbidities included bilateral mild nuclear sclerotic cataracts. Visual acuities at baseline were 20/25 + 2 in the right eye and 20/40 in the left eye. The baseline MMI of the right eye including FA (not shown) demonstrated type 1 NV with overlying subretinal and intraretinal fluid and two small juxtafoveal spots of GA in the right eye and a few extramacular drusen. Intermediate sized, refractile drusen and 4 areas of GA were seen in the left eye (Fig. 7). Over the following 95 months, the right eye received 76 intravitreal anti-VEGF injections on a TER with injections given approximately every 5–7 weeks. At the most recent follow-up, visual acuity was 20/30 − 2 in the right eye and 20/200 − 1 in the left eye. The area of GA at baseline visit was 0.56 mm^2^ for the right eye and 4.62 mm^2^ for the left eye. The area of GA at final visit was 0.61 mm^2^ for the right eye and 7.84 mm^2^ for the left eye (Fig. 8). The rate of GA progression was 0.00052 mm^2^/month (or 0.00065 mm^2^/anti-VEGF injection) on the right eye and 0.03389 mm^2^/month on the left eye.

## Discussion

The relationship between neovascular subtypes, anti-VEGF dosing regimen and the development of GA in AMD is complex [7, 11, 12]. Although anti-VEGF therapy has become the mainstay treatment for neovascular AMD, there have been some concerns regarding the long-term effects of these agents on outer retinal structures. Recent analyses of GA data from the CATT and IVAN studies have suggested that more frequent dosing of anti-VEGF agents may be associated with an increased risk of new GA and a greater rate of GA progression [8, 13]. Lois et al. monitored GA progression using a similar methodology to our series and concluded that each additional injection of anti-VEGF increased the odds of GA development by a factor of 1.35 [14]. To date however, a difference in the rate of GA progression between ranibizumab and bevacizumab has not been proven [13].

The prospective study performed by Xu and colleagues demonstrated that the risk of GA progression has important correlations to the subtype of NV that is being treated. Upon analyzing the risk factors associated with the development of GA, Grunwald et al. [8] observed that RAP lesions were associated with a 1.69 greater risk of GA development, a finding that supports a previous report from McBain et al. [11]. Analysis of data from the CATT study also revealed that the presence of intraretinal fluid was associated with a two-fold risk of GA formation, while an increase in subretinal fluid and sub-RPE tissue thickness conferred a decreased risk of GA formation [8]. One could speculate that the presence of intraretinal fluid is not a risk factor but rather a consequence of irreversible damage to the outer retina. Our findings, however, suggest that the presence of neovascular tissue in the sub-RPE space may confer a protective effect against the development of GA. It is plausible that proliferation of vascular tissue adjacent to the retinal pigment epithelium, although pathologic, may serve to nourish the RPE layer and thus preserve outer retinal structures.

In AMD, development of neovascularization in one eye is a strong predictor for the development of NV in the fellow eye [15]. The risk of the fellow eye developing NV is also related to the duration of AMD. Therefore, it is uncommon for patients with AMD with NV in one eye not to have NV of the fellow eye after a follow-up period of almost 10 years. For this reason, the patients included in this case series represent an ideal experimental model to study AMD because the pathogenic factors that modulate the course of this disease, including genetics, environmental exposure and diet, are expected to have an equal influence on the non-neovascular and neovascular eyes of the same patient. Patients included in this case series, therefore, allow us to assess the temporal relationship between the phenotypic manifestations of AMD, the iatrogenic influence of intraocular therapies and the risk of GA progression in the absence of other confounding factors.

Work by Sunness and colleagues and also the results of the CATT study have shown that there is strong concordance in the rate of GA development between eyes of the same patient [16, 17]. The significant disparity in the rate of GA development in this series, which was found to be statistically significant, would suggest that a pathogenic mechanism that was unique to the neovascular eye was reducing the rate of GA progression. This finding is inconsistent with previous reports that found the risk of GA progression to be positively correlated with the number of anti-VEGF injections [13, 16]. The mean number of anti-VEGF injections per eye in this series was 65 and one would expect an increased rate of GA progression in the eyes receiving treatment. However, we observed the converse effect. Taken together, these findings suggest that type 1 neovascular tissue may serve a protective role and might reduce the rate of GA progression in AMD.

## Conclusions

Currently, there remains some debate concerning the most efficacious treatment strategy for the management of neovascular AMD [18]. The observations in this small series support previous speculations that type 1 NV may reduce the risk of GA progression. From a clinical standpoint, it may therefore be advantageous to have neovascular tissue in the sub-RPE space, and regimens that aim to achieve total regression of type 1 NV may be associated with greater visual morbidity in the long-term. We acknowledge that more quantitative data is necessary to validate these findings.

## Abbreviations

GA: geographic atrophy
AMD: age-related macular degeneration
RPE: retinal pigment epithelium
FA: fluorescein angiography
RAP: retinal angiomatous proliferation
CATT: comparison of AMD treatment trials
IVAN: inhibition of VEGF in age-related choroidal neovascularization
VEGF: vascular endothelial growth factor
NV: neovascularization
MMI: multimodal imaging
AF: autofluorescence
SD-OCT: spectral-domain optical coherence tomography
TER: treat-and-extend-regimen
